# Apoptotic Effects of *Xanthium strumarium* via PI3K/AKT/mTOR Pathway in Hepatocellular Carcinoma

**DOI:** 10.1155/2019/2176701

**Published:** 2019-11-07

**Authors:** Juyoung Kim, Kyung Hee Jung, Hyung Won Ryu, Doo-Young Kim, Sei-Ryang Oh, Soon-Sun Hong

**Affiliations:** ^1^Department of Biomedical Sciences, College of Medicine, Inha University, 3-ga, Sinheung-dong, Jung-gu, Incheon 400-712, Republic of Korea; ^2^Natural Medicine Research Center, Korea Research Institute of Bioscience and Biotechnology, Cheong-ju si, Chungcheongbuk-do 28116, Republic of Korea

## Abstract

*Xanthium strumarium* (XS) has been traditionally used as a medicinal herb for treating inflammatory diseases, such as appendicitis, chronic bronchitis, rheumatism, and rhinitis. In this study, we yielded ethanol extracts from XS and investigated whether they could inhibit the progression of hepatocellular carcinoma (HCC) and its underlying mechanism. The XS-5 and XS-6 extracts dose-dependently inhibited the growth and proliferation in HCC cell lines. The apoptotic effects of them were observed via increased levels of cleaved caspase-3 and cleaved PARP, as well as elevated numbers of terminal deoxynucleotidyl transferase-mediated dUTP-biotin end labeling- (TUNEL-) positive apoptotic cells. They also decreased XIAP and Mcl-1 expression via loss of mitochondrial membrane potential. Additionally, they inhibited the invasion and migration of HCC cells. In an *ex vivo* model, the extracts significantly inhibited tumor cell growth and induced apoptosis by increasing the expression of the cleaved caspase-3. A mechanistic study revealed that they effectively suppressed PI3K/AKT/mTOR signaling pathways in HCC cells. Taken together, our findings demonstrate that they could efficiently not only induce apoptosis but also inhibit cell growth, migration, and invasion of human HCC cells by blocking the PI3K/AKT/mTOR pathway. We suggest XS-5 and XS-6 as novel natural anti-HCC agents.

## 1. Introduction

Hepatocellular carcinoma (HCC) is the fifth commonest malignancy and the third commonest cause of cancer mortality [1]. Most of patients with HCC have a poor prognosis because detection of the disease usually occurs at an advanced stage. Patients diagnosed with HCC have a very low survival rate, with about 9% of them surviving for 5 years or less after diagnosis [2]. Despite considerable advances in HCC diagnosis and treatment, the proportion of resectable HCC tumors and cases amenable to liver transplantation remains low. Additionally, chemotherapy and radiotherapy offer limited benefits and are associated with severe adverse effects. To date, there are no effective curative methods due to the high invasion, early metastasis, and unexpected high recurrence rates of HCC after surgery or interventional treatments such as transcatheter arterial chemoembolization (TACE) [3]. Therefore, it is important to explore alternative strategies that may effectively control HCC. Presently, there are a lot of interests in traditional medicines, which is used for cancer monotherapy or in combination with other cancer treatments.

Plant extracts have been used for their medicinal properties, and their active substances form the basis of herbal medicines that have been practiced for a long time and still provide treatments for humankind. Among the plants in the genus *Xanthium* (Family Asteraceae), *X. strumarium* (XS) has traditionally been used as herbal medicine in Indo-China, Malaysia, America, and Europe [4]. The entire plant has been used as a medicine to cure headache, arthritis, rhinitis, and other ailments which supports its traditional medicinal usage in inflammatory diseases [4]. Also, XS contains many active compounds, including glycosides, phytosterols, phenolic acids, and xanthiazone, which have shown antibacterial, antifungal, hypoglycemic, and cytotoxic properties [5, 6]. Recently, ethanol, dichloromethane, and chloroform extracts of XS have exhibited *in vitro* cytotoxic activities against various cancer cell lines [7, 8]. Despite the important body of work that has been performed on XS, the cellular and molecular mechanisms underlying the anticancer actions of this plant remained poorly characterized.

In this study, we obtained various ethanol extracts of XS through an optimized extraction process. Among these extracts, XS-5 and XS-6 were selected as the most effective and were investigated for their anticancer activity and mechanism of action in HCC. Our study revealed that XS-5 and XS-6 significantly induced apoptosis and inhibited cell proliferation by inhibiting the PI3K/AKT/mTOR pathway in HCC.

## 2. Materials and Methods

### 2.1. Plant Material

*X. strumarium* fruits were collected from the Inner Mongolia Autonomous Region, China (Lot No. K1451201707), in July 2017, and were identified by Dr. Hocheol Kim. A voucher specimen (D180305001) of this raw material was leaved in the Herb Resource Bank of Traditional Korean Medicine (http://herb-bank.com) at Kyung Hee University in Seoul, Korea. The fruits were roasted using a method described in Chinese Pharmacopeia as follows: the fruits were stir-fried for 1 h in a kitchen stove at 180 ± 5°C until the fruit surface turned dark brown [9].

### 2.2. Preparation of *Xanthium strumarium* Extracts

The processed fruits were freeze-dried and cut into small pieces with a laboratory blade cutter. The powdered samples (2.0 kg) were extracted with 70% ethanol (3 L × 3) using an SD 300H sonicator (SD-ultra, Seoul, Korea) at 40 KHz (15 min each). The organic extracts were concentrated after combination *in vacuo* at 40°C to produce a dried extract (126.5 g, 6.3%). The extracts (4.5 g) were subjected to medium-pressure liquid chromatography (Spot Prep II 250 Armen, Paris, France) with a reversed-phase silica gel column (YMC ODS-AQ, 10 *μ*m, 220 g) using a stepwise MeOH-H_2_O gradient (0–5 min 20% MeOH, 5–35 min 20–80% MeOH, 35–55 min 80–100% MeOH, 55–80 min 100% MeOH, 30 mL/min, 80 min) to give 7 fractions (XS 1–7). This fractionation process was repeated 16 times to produce a large quantity of each fraction for further separation and biological evaluation. XS-5 and XS-6 fractions of peak-based collection were eluted at 95–100% (50–60 min) and 100% (60–80 min) gradient of MeOH condition, respectively.

### 2.3. Cells and Materials

HCC cells (Huh-7 and Hep3B) were purchased from the Japanese Cancer Research Resources Bank (JCRB) (Shinjuku, Japan) and cultured in Dulbecco Modified Eagle Medium (DMEM) containing 10% fetal bovine serum (FBS) and 1% antibiotics. Cells were maintained under an optimal condition (37°C, 5% CO_2_ and humidified atmosphere) in a CO_2_ incubator. Cell culture materials, FBS, and penicillin/streptomycin were purchased from Invitrogen (Carlsbad, CA).

### 2.4. Cell Viability Assay

The viability of XS-treated cells was analyzed using a 3-(4, 5-dimethylthiazol-2-yl)-2, 5-diphenyl tetrazolium bromide (MTT) assay. In brief, Hep3B and Huh-7 cells (6 × 10^3^/well) were seeded in 96-well plates and incubated for 24 h. The cells were treated with various concentrations of XS (1, 5, 10, 100, and 500 *μ*g/ml). After incubation for 72 h, MTT solution was added to each well for another 4 h, and plates was incubated at 37°C. The formazan crystals that formed in cells were dissolved in dimethyl sulfoxide by constant shaking for 10 min. The plate was read immediately on an automatic microplate reader with UV absorbance detection at 540 nm. Each experiment was set in triplicate and performed three times independently.

### 2.5. Western Blot Analysis

For western blotting, cells were lysed buffer including 1% Triton X-100 and 1% NP-40, as well as the following protease and phosphatase inhibitors. The first step is to separate the proteins by sodium dodecyl sulfate-polyacrylamide gel electrophoresis (SDS-PAGE), and then, proteins can be electrophoretically transferred to PVDF membranes. The membranes were immunostained with primary antibodies. Furthermore, HRP-conjugated secondary antibodies are added. Detection was carried out using an enhanced chemiluminescence reagent (Amersham Biosciences, Buckinghamshire, UK). Antibodies against cleaved PARP, cleaved caspase-3, XIAP, Mcl-1, p-mTOR, p-AKT (Tyr308), p-4EBP1, p-GSK3*β*, and *β*-actin were purchased from Cell Signaling Technology (Danvers, MA), Abcam (Cambridge, MA), and Santa Cruz Biotechnology (Santa Cruz, CA).

### 2.6. 5′-Bromo-2′-Deoxyuridine (BrdU) Cell Proliferation Assay

Hep3B and Huh-7 cells (7 × 10^3^ per well) were incubated with various concentrations of XS-5 and XS-6 in 96-well plates at 37°C for 6 h. Following incubation, an evaluation of cell proliferation was performed by the measurement of BrdU incorporation into newly synthesized cellular DNA by utilizing a BrdU cell proliferation assay kit (Merck, Darmstadt, Germany) according to the manufacturer's instructions. Briefly, cells were incubated with 5 *μ*M BrdU for 3 h. After incubation, fixing/denaturing solutions were supplied for 30 min at room temperature. Detection antibody solution of 100 *μ*l was incubated for 1 h at room temperature. After incubation of HRP-conjugate solution, TMB substrate was incubated for 15–30 min. Stop solution of 1 mM H_2_SO_4_ was added, and this reaction was measured at 450 nm by a microplate reader. All tests were carried out for two sample replications.

### 2.7. Terminal Deoxynucleotidyl Transferase-Mediated Nick End Labeling (TUNEL) Assay

Hep3B and Huh-7 cells (5 × 10^3^) were seeded onto an 18 mm cover glass and added culture media. After 24 h incubation, cells were treated with various concentrations of XS-5 and XS-6 for 24 h and then fixed in a mixture of ice-cold acetic acid and ethanol (1 : 2) for 5 min and washed with PBS. For apoptotic cells detection, terminal deoxynucleotidyl transferase-mediated nick end labeling (TUNEL) kit was used (Chemicon, Temecula, CA), following the manufacturer's instructions. All tests were carried out for two sample replications.

### 2.8. Analysis of Mitochondrial Membrane Potential

The JC-1 mitochondria staining kit (JC-1, Grand Island, NY) was used to measure mitochondrial membrane potential. Fluorescent dye accumulation in mitochondria can display high potential-dependent accumulation in mitochondria. Hep3B and Huh-7 cells were seeded into 18 mm cover glasses. After attachment, the cells were treated with XS-5 and XS-6 (100 *μ*g/ml) for 6 h and added with 100 *μ*l of JC-1 solution (final concentration of 12.5 *μ*g/ml) at 37°C for another 30 min. 4, 6-Diamidino-2-phenylindole (DAPI) was added to visualize the nuclei. After covering with mounting solution, cells were viewed with a confocal laser scanning microscope (Olympus, Tokyo, Japan). All tests were carried out for two sample replications.

### 2.9. Detection of Cytochrome *c* Location

Hep3B and Huh-7 cells were treated with XS-5 and XS-6 (100 *μ*g/ml) for 6 h. A mitochondrion-specific FITC dye (Molecular Probes Inc, Eugene, OR) was incubated for 1 h. For cell fixation, an acetone-methanol solution (1 : 1) was added for 5 min at −20°C. The fixed cells were incubated with cytochrome *c* antibody (Santa Cruz Biotechnologies, Santa Cruz, CA) overnight at 4°C. A mouse fluorescence-labeled secondary antibody was incubated (1 : 100, Dianova, Hamburg, Germany) for 1 h after counter staining with DAPI solution. After covering with mounting solution, cells were viewed with a confocal laser scanning microscope (Olympus). All tests were carried out for two sample replications.

### 2.10. Migration

Huh-7 cells were seeded in a 60 mm dish at 90% confluence and scratched with micropipette tips. The cells that peeled off were removed using PBS, and the wounded Huh-7 cells were treated with XS-5 and XS-6 (100 *μ*g/ml) for 48 h. The wounded cells were washed with PBS and then were fixed with methanol. All tests were carried out for two sample replications.

### 2.11. Invasion Assay

For the invasion assay, 24-well modified Boyden chambers (pore size, 8 *μ*m) were coated with 10% Matrigel. Next, 2 × 10^5^ Huh-7 cells with or without 100 *μ*g/ml of XS-5 and XS-6 were placed in the upper chamber, and the lower chamber was filled with 750 *μ*L of completed media. After 48 h of incubation, the cells that reached the lower surface in 4% paraformaldehyde for 20 min were stained with 0.5% crystal violet. The cells on the upper surface of the filter were removed with cotton swab and counted under a magnification of 400X. We chose five random fields and counted the number of invaded cells. All tests were carried out for two sample replications.

### 2.12. Immunofluorescence Microscopy

The cells were seeded at a density of 2 × 10^5^ cells/well onto a 12-well plate in DMEM. Next day, cells were treated with XS-5 and XS-6 (100 *μ*g/ml) for 6 h. After fixation with an acetic acid-ethanol (2 : 1) solution, blocking solution with 5% goat and horse serum/PBS was incubated to block nonspecific binding for 1 h. Primary antibodies were incubated at 4°C overnight, and fluorescein-labeled secondary antibody was subsequently added for 1 h. The nuclei counter staining were conducted with DAPI for 30 min. After covering with mounting solution, slides were viewed with a confocal laser scanning microscope (Olympus, Tokyo, Japan). All tests were carried out for two sample replications.

### 2.13. Immunohistochemistry

After fixing the tissue and embedding it in paraffin, we performed immunohistochemical staining using 8 *μ*m-thick sections. Heat-induced epitope retrieval (HIER) was performed in a citrate buffer (pH 6.0) for 10 min, and then, peroxidase was quenched with 3% hydrogen peroxide (H_2_O_2_) in PBS for 8 min. The sections were blocked with blocking solution including normal goat or horse serum for 40 min. Next, the sections were incubated overnight with anti-proliferating cell nuclear antigen (PCNA) (Abcam) and cleaved caspase-3 (Cell Signaling Technologies) at 4°C. After being washed with PBS several times, the sections were also incubated with biotinylated secondary antibodies for 1 h and then streptavidin-HRP was applied. Finally, the tissue section was developed with a diaminobenzidine tetrahydrochloride (DAB) and counterstained with hematoxylin. More than 3 random fields of each section were evaluated at a 400X magnification. All tests were carried out for two sample replications.

### 2.14. *Ex Vivo* Organotypic Tumor Spheroid Assay

Male BALB/c nude mice (20–25 g) were gained from Orient Bio. Animal Inc. (Seoul, Republic of Korea). BALB/c nude mice were kept under standard laboratory conditions as acclimatization period for 1 week (23°C, 55 ± 5% humidity and a 12 h light/dark cycle) and maintained with free access to water and a standard diet ad libitum. The animals were housed in groups of four in 595 × 380 × 200 mm cages and were monitored twice daily for health and clean behavior. No adverse events were observed. Animal studies were performed in accordance with the guidelines of the INHA Institutional Animal Care and Use Committee (INHA IACUC) at Inha University (approval ID : INHA 180523–569). Total Huh-7 8 × 10^6^ cells were inoculated by subcutaneously injection into 6-week-old male nude mice (Orient-Bio, Korea). When the tumor size reached approximately 300–500 mm^3^, they were randomly selected and surgically removed (*n* = 6). A 2 mm diameter section was excised and explanted on 2% agarose-coated 24-well plates with culture medium at 37°C. The mice were anesthetized by intraperitoneal injection of xylazine-ketamine anesthetics (ketamine 100 mg/ml, 100 mg/kg; xylazine 2% 20 mg/ml, 20 mg/kg). For xylazine-ketamine anesthetics, 1 ml ketamine was mixed with 1 ml xylazine and 8 ml saline was added. A xylazine-ketamine anesthetic of 0.2 ml was injected to every mouse. Fresh culture medium was changed every 2 or 3 days for 2 weeks. When explants became spherical (i.e., spheroids, 2 mm diameter), we used for subsequent experiments.

### 2.15. Chromatographic Conditions of HPLC-MS Analysis

To acquire chromatograms, we used an Agilent 1100 series high-performance liquid chromatography (HPLC) system (Agilent Corp., Santa Clara, CA). All the chromatographic experiments were analyzed on a Phenomenex Kinetex C18 column (100 mm × 4.6 mm i.d. 2.6 *μ*m). The LC mobile phase was constituted by 0.1% formic acid and 0.1% formic acid in methanol. The conditions of solvent gradient elution were 30% for 0–2 min, 30–90% for 2–12 min, 90% for 12–22 min, 90–30% for 22–22.1 min, and 30% for 22.1–30 min, at a flow rate of 0.5 ml/min. Temperature of column was kept at 40°C, and sample solutions of 2 *μ*l were fixedly injected. Also, the eluent was injected to an ESI-LTQ-XL-Linear Ion Trap mass spectrometer (Thermo Scientific), and all data were obtained in full-scan and positive modes, with a mass range from 100 to 800 m/z.

### 2.16. Statistical Analysis

Data are expressed as the mean ± standard deviation (SD) and were analyzed by unpaired Student's *t*-tests, followed by one-way ANOVA followed by the Tukey test or two-way.

ANOVA followed by the Bonferroni test was used. Statistical analysis was performed with Graph Pad PRISM (Version 5.0). A *p* value of 0.05 or less was considered statistically significant.

## 3. Results

### 3.1. XS-5 and XS-6 Inhibited the Growth and Proliferation of HCC Cells

**Figure 1:**
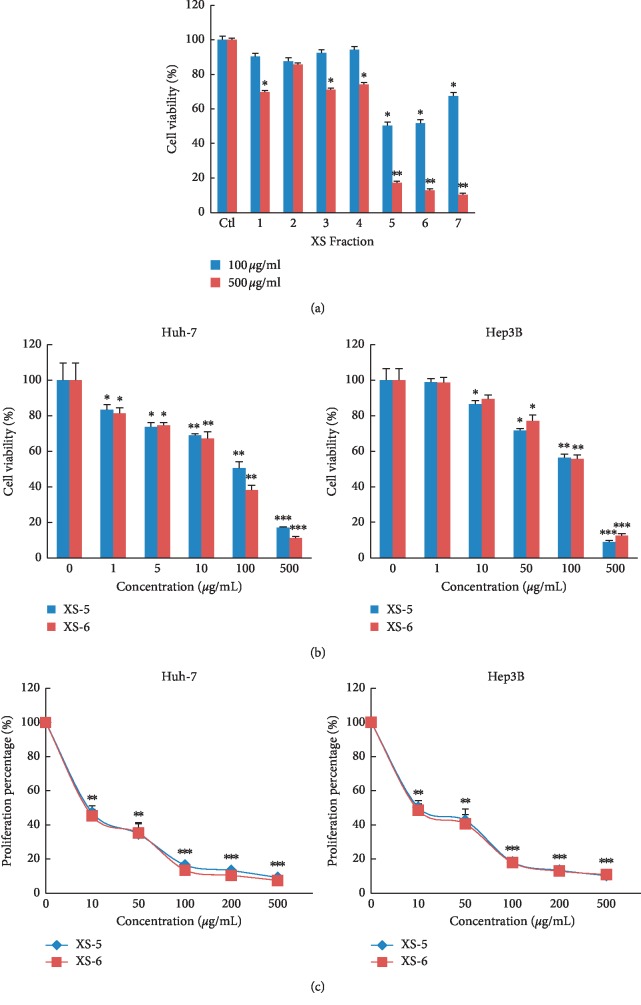
Effects of XS-5 and XS-6 on the growth and proliferation of HCC cells. (a) Cytotoxic effects of XS extracts on Huh-7 cells were measured using an MTT assay. (b) Hep3B and Huh-7 cells were treated with XS-5 and XS-6 at the indicated concentration for 72 h, and the cytotoxic effects were measured with an MTT assay. (c) Proliferation of Hep3B and Huh-7 cells was assessed using a BrdU proliferation assay. Results are expressed as percentage of cell proliferation relative to that of the control. Data are represented as means ± SD of triplicates (^*∗*^*p* < 0.05, ^*∗∗*^*p* < 0.005, and ^*∗∗∗*^*p* < 0.001*vs* control).

The MTT assay revealed XS-5 and XS-6 to be the most effective cytotoxic agents among the XS extracts (Figure 1(a)), reducing the viability of both Hep3B and Huh-7 cells in a dose-dependent manner (Figure 1(b)). In particular, the mean IC_50_ values of XS-5 and XS-6 were 100 *μ*g/mL. For this reason, we chose this dose for further experiments. Interestingly, XS-5 and XS-6 fractions, which were isolated using a stepwise MeOH-H_2_O gradient after extraction by 70% methanol, showed about 2-fold cytotoxic effect compared with XS-1 fraction (traditional extraction method using 70% methanol, Figure 1(a)). Next, to identify the effects of XS-5 and XS-6 on cell proliferation, we measured BrdU incorporation into DNA. XS-5 and XS-6 inhibited BrdU incorporation in a dose-dependent manner (Figure 1(c)).

### 3.2. XS-5 and XS-6 Induced Apoptotic Cell Death

**Figure 2:**
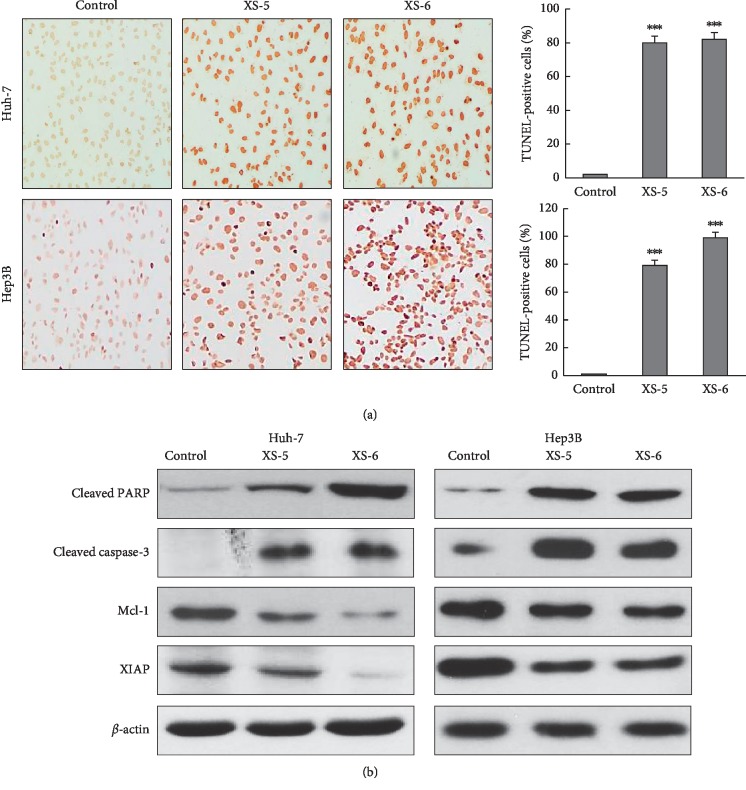
Effects of XS-5 and XS-6 on apoptosis of HCC cells. (a) Hep3B and Huh-7 cells were treated with XS-5 and XS-6 (100 *μ*g/ml) for 24 h, and the TUNEL assay was performed and photographed at 400X magnification. (b) The expressions of cleaved caspase-3, PARP, XIAP, and Mcl-1 were determined by western blot analysis in cells treated with XS-5 and XS-6 (100 *μ*g/ml) for 48 h. Data are represented as means ± SD from triplicate experiments (^*∗∗∗*^*p* < 0.001*vs* control).

To examine the apoptotic effects of XS-5 and XS-6, we performed TUNEL staining in Hep3B and Huh-7 cells. When treated with XS-5 and XS-6 (100 *μ*g/mL) for 24 h, the cells were shown features of apoptotic cell death, such as DNA fragmentation. As shown in Figure 2(a), there was a higher percentage of TUNEL-positive cells in XS-5- and XS-6-treated groups. Western blot analysis after XS-5 and XS-6 treatment for 24 h revealed increased expression of cleaved caspase-3 and PARP and decreased expression of XIAP and Mcl-1 (Figure 2(b)). These findings showed that XS-5 and XS-6 inhibited cell proliferation through the induction of apoptosis in HCC cells.

### 3.3. XS-5 and XS-6 Induced Mitochondrial-Dependent Apoptosis in HCC Cells

**Figure 3:**
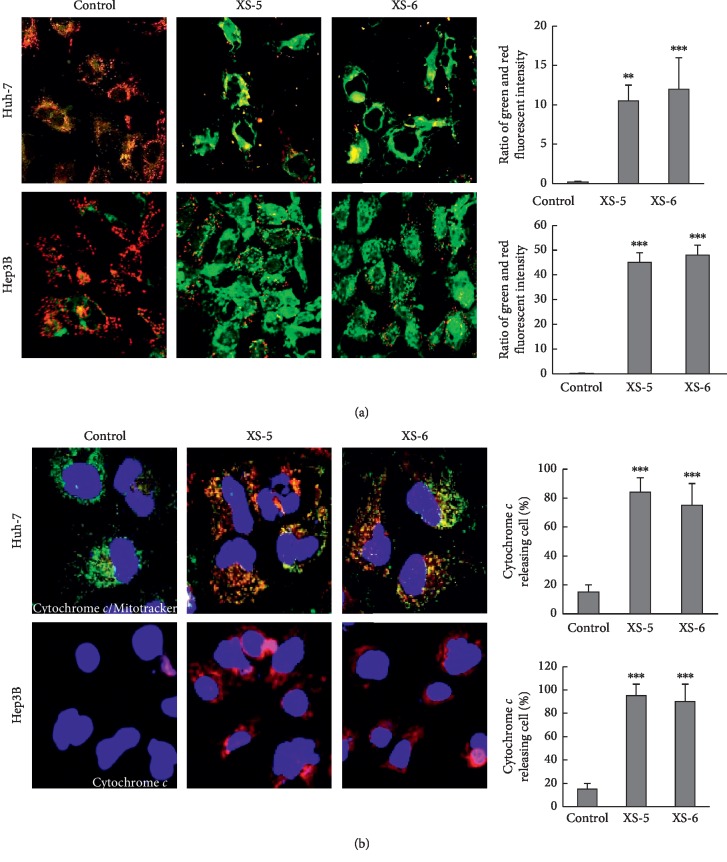
Effects of XS-5 and XS-6 on mitochondrial apoptosis in HCC cells. (a) When Hep3B and Huh-7 cells were treated with XS-5 and XS-6 (100 *μ*g/ml) for 6 h, the mitochondrial membrane potential was determined by JC-1 staining and analyzed with an Olympus confocal laser scanning microscope. The results (green : red ratio) are expressed as the percentage of cells treated with XS-5 and XS-6. (b) Hep3B and Huh-7 cells were treated with XS-5 and XS-6 (100 *μ*g/ml) for 6 h and were stained with Mitotracker (green) and cytochrome *c* (red). Localization of cytochrome *c* in the cytosol was photographed at 400X magnification. Data are expressed as the means ± SD from triplicate experiments (^*∗∗*^*p* < 0.005 and ^*∗∗∗*^*p* < 0.001*vs* control).

We used JC-1 staining to assess the effects of XS-5 and XS-6 on changes in mitochondrial membrane potential, which correlate with intrinsic apoptosis. As shown in Figure 3(a), the cytosol of control cells was observed by heterogeneous staining with both red and green components of JC-1 fluorescence. Consistent with mitochondrial localization, red fluorescence was highly exhibited in granular structures dispersed throughout the cytosol. XS-5 and XS-6 treatments induced marked changes in mitochondrial membrane potential, as evidenced by the disappearance of red fluorescence or increased amounts of green fluorescence in most cells. It is well known that membrane potential of mitochondria induces the cytochrome *c* release into the cytosol.  Figure 3(b) depicts our observation that XS-5 and XS-6 increased cytochrome *c* release together with a decrease in the colocalization of cytochrome *c* and mitochondria, relative to the control.

### 3.4. XS-5 and XS-6 Suppressed Invasion and Migration of HCC Cells

**Figure 4:**
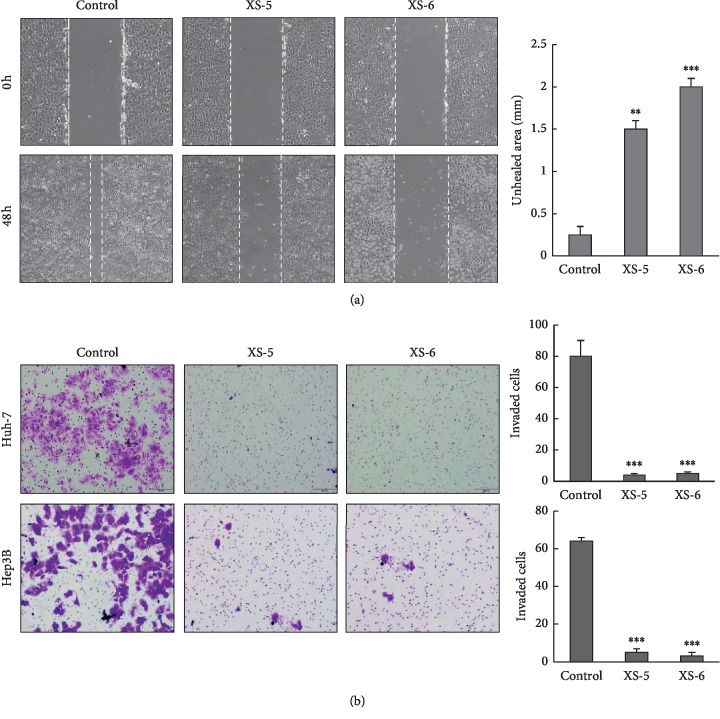
Effects of XS-5 and XS-6 on migration and invasion of HCC cells. (a) Representative images of a wound healing assay in which Huh-7 cells were treated or not treated with XS-5 and XS-6 (100 *μ*g/ml) for 24–48 h. All images were captured at 200X magnification. (b) Cell invasion assay was performed using Matrigel-coated transwells. Cells were treated or not treated with XS-5 and XS-6 (100 *μ*g/ml) for 48 h and stained with 0.5% crystal violet. Images were captured at 200x magnification. The number of invaded cells was presented as the mean ± SD from triplicate experiments (^*∗∗*^*p* < 0.005 and ^*∗∗∗*^*p* < 0.001*vs* control).

It has been shown that cancer metastasis is associated with cell invasion and migration [10]. To assess the effects of XS-5 and XS-6 on the migration of HCC cells, we seeded Huh-7 cells in 6-well plates and grew them until confluence reached 90%. As shown in Figure 4(a), in the wound healing cell migration assay, control cells were healed up to 90% of the wound area within 48 h, but the cells treated with XS-5 and XS-6 significantly inhibited the migration of Huh-7 HCC cells. As the invasive property of cancer cells is necessary for the early steps of metastasis, we next examined the effects of XS-5 and XS-6 on the invasion of HCC cells using transwell 24-unit invasion assays (Figure 4(b)). Similar to the results of the migration analysis, cellular invasion was effectively inhibited by the XS treatments.

### 3.5. XS-5 and XS-6 Inhibited the PI3K Pathway in HCC Cells

**Figure 5:**
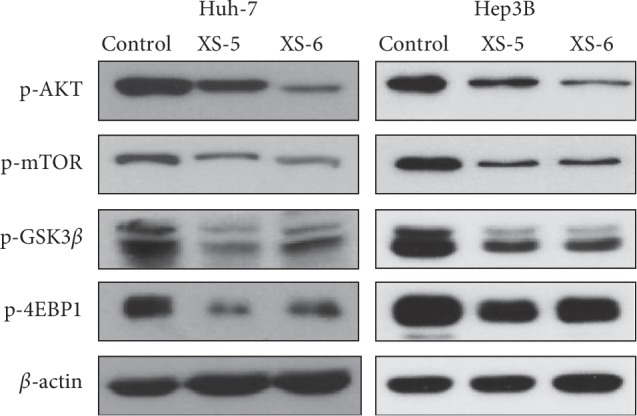
Effects of XS-5 and XS-6 on the PI3K/AKT signaling pathway of HCC cells. The effects of XS-5 and XS-6 on the expression levels of AKT, mTOR, GSK3*β*, and 4EBP1 and their phosphorylated forms were determined by western blot analysis. Hep3B and Huh-7 cells were treated with XS-5 and XS-6 (100 *μ*g/ml) for 6 h.

Aberration of the PI3K/AKT/mTOR pathway which is a cell survival pathway is associated with HCC carcinogenesis [11]. Therefore, we investigated whether XS-5 and XS-6 could inhibit the PI3K/AKT signaling pathway in HCC cells. After Hep3B and Huh-7 cells were treated with XS-5 and XS-6 (100 *μ*g/mL) for 6 h, the expression of PI3K/AKT signaling-related molecules was determined by western blot analysis. As shown in Figure 5, XS-5 and XS-6 reduced the expressions of p-AKT, p-mTOR, p-GSK3*β*, and p-4EBP1, downstream signals of the PI3K/AKT pathway.

### 3.6. XS-5 and XS-6 Inhibited Cell Proliferation and Induced Apoptosis in *Ex Vivo* HCC Primary Tumor Organotropic Spheroids

**Figure 6:**
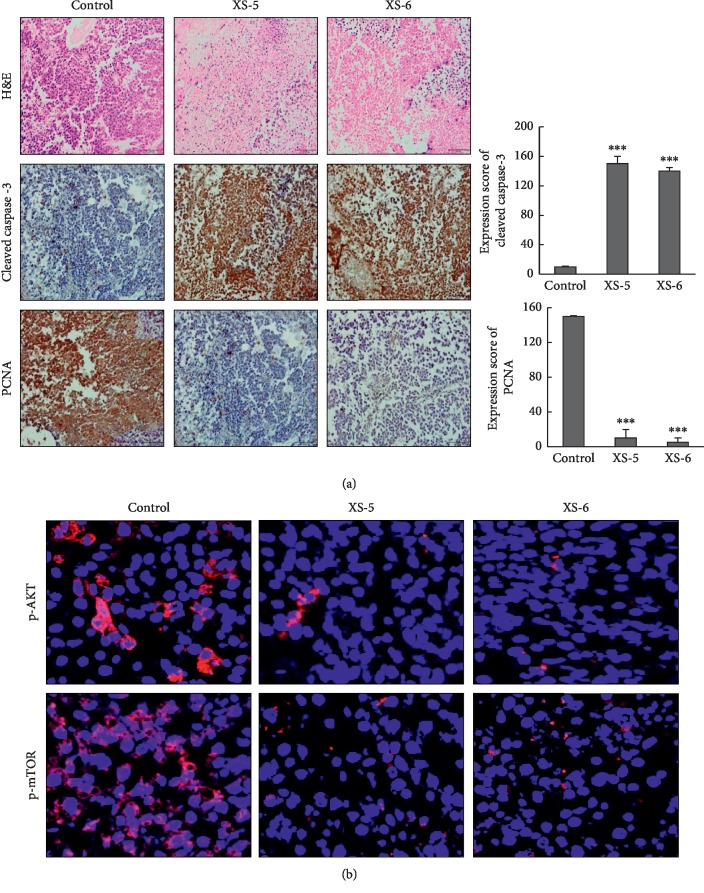
Effects of XS-5 and XS-6 in HCC *ex vivo* tumor models. (a) Balb/c nude mice bearing Huh-7 HCC xenograft tumors were cut into small pieces of ∼2 mm, and each piece of tumor was maintained in culture media. Tumor spheroids cultured from xenograft tumor tissues were treated with XS-5 and XS-6 (100 *μ*g/ml) for 5 days. Immunostaining and hematoxylin and eosin staining for PCNA and cleaved caspase-3 were carried out. (b) Tumor spheroids were excised and processed for immunofluorescence for p-AKT and p-mTOR. Images were captured at 400X magnification. Data are represented as the mean ± SD (^*∗∗∗*^*p* < 0.001*vs* control).

To identify the therapeutic effects of XS-5 and XS-6, we performed an organotropic tumor spheroid assay using mouse xenograft tumor tissues, which comes close to *in vivo* physiologic characteristic, such as physical barriers to drug transport and multicellular architecture. From hematoxylin and eosin staining, we observed that XS-5 and XS-6 treatments induced significantly greater tumor cell apoptosis and necrosis than control Huh-7 tumor spheroids. The apoptotic effect was also identified via the increased expression of the cleaved caspase-3 and decreased expression of the cell proliferation marker PCNA in HCC tumor spheroids (Figure 6(a)). Additionally, XS-5 and XS-6 decreased expression of p-AKT and p-mTOR (Figure 6(b)). Collectively, these findings revealed that XS-5 and XS-6 have potent antitumor efficacy in *ex vivo* HCC tumor organotropic spheroids.

### 3.7. Constituents of XS-5 and XS-6 Were Analyzed by HPLC-Mass Spectrometry

**Figure 7:**
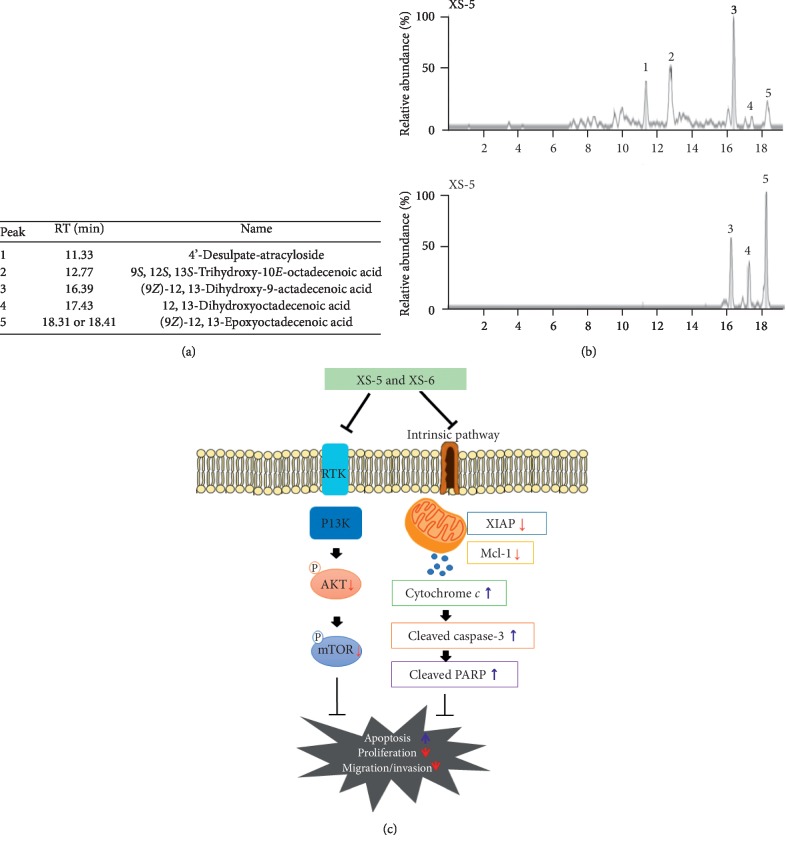
High-performance liquid chromatography-mass spectrometry chromatogram fingerprinting of XS-5 and XS-6. (a) Peaks of XS-5 and XS-6 were deduced based on comparing individual peak retention times with those of the authentic reference substance and verified by LC-MC. (b) Scheme for how XS-5 and XS-6 induce apoptosis and inhibit the growth of HCC cells.

The high anticancer potencies of XS-5 and XS-6 compelled the execution of UPLC-QTof-MS (Micromass Q-Tof Premier™, Waters Corporation, Milford, MA) analyses using an ACQUITY BEHC18 chromatography column (2.1 × 100 mm, 1.7 *μ*m) with a linear gradient (0 min, 1% B; 0–1.0 min, 1–5% B; 1.0–10.0 min, 5–30% B; 10.0–17.0 min, 30–60% B; 17.0–17.1 min, 60–100% B, 17.1–19.0 min, 100%B, 19.0–20.0 min, back to 1% B) of acetonitrile/water (HPLC-grade, Merck, Darmstadt, Germany). After analysis, major compounds were characterized using mass data (experimental *m/z*, HRESIMS, error ppm, molecular formulae). As shown in Figure 7, our experiments revealed that the deprotonated molecule [M-H]^−^ was the most abundant for five compounds from XS-5 and XS-6: peak 1 (*m/z* 645, C_30_H_46_O_13_S_1_, 4′-desulphate-atractyloside), peak 2 (*m/z* 329, C_18_H_34_O_5_, 9*S*, 12*S*, 13*S*-trihydroxy-10*E*-octadecenoic acid), peak 3 (*m/z* 313, C_18_H_34_O_4_, (9*Z*)-12, 13-dihydroxy-9-octadecenoic acid), peak 4 (*m/z* 315, C_18_H_36_O_4_, 12, 13-dihydroxyoctadcenoic acid), and peak 5 (*m/z* 295, C_18_H_32_O_3_, (9*Z*)-12, 13-epoxyoctadecenoic acid). Five compounds were tentatively identified by comparison for publishing MS results using Reaxys and online databases such as PubChem and ChemSpider [12].

## 4. Discussion

Natural products have played a key role in drug discovery and development. They provide a source and inspiration for developing effective treatments for human diseases [13, 14]. Many natural products are currently being applied as cancer treatments and alternative medicines, supplements, or nutraceuticals to alleviate the side effects of existing anticancer drugs. Recent studies have shown that herbal medicines or their ingredients, which are important parts of complementary and alternative medicine for cancer, are widely used for preclinical and clinical research [15]. In particular, some natural herbal medicines have been used to treat HCC [16].

Numerous studies have reported the pharmacological efficacy and benefits of XS against various inflammatory diseases, such as allergic rhinitis, rheumatism, and sinusitis [17]. Although the anti-inflammatory effects of XS have been extensively studied, to our knowledge, there are no published studies on the anticancer effects of XS or its underlying mechanism against HCC. Therefore, we yielded various ethanol extracts of the aerial parts of XS and focused on XS-5 and XS-6, which had the most potent antiproliferative effects. Our study revealed that XS-5 and XS-6 inhibited cell growth and induced mitochondria-mediated apoptosis by blocking the PI3K/AKT signaling pathway in HCC. To our knowledge, the anticancer effects of XS are not well documented, and this is the first study to demonstrate the anticancer effects of XS in HCC.

Apoptosis is, evolutionarily, a highly conserved intrinsic death program that plays a key role in maintaining tissue homeostasis during development [18]. Consequently, too little apoptosis can promote tumorigenesis even without an increase in proliferation. In the context of cancer, natural products can modulate apoptosis signaling pathways [19]. Unlike pharmaceutical drugs, natural extracts induce apoptosis by multiple cellular signaling pathways that are frequently deregulated in cancers without cytotoxicity, resulting in efficacious killing of cancer cells. For these reasons, we supposed that XS extracts might provide novel findings for cancer treatment by induction of apoptosis in HCC. We demonstrated DNA fragmentation in HCC cells treated with XS-5 and XS-6. Additionally, the observations of caspase-3 activation and PARP cleavage confirmed that the promotion of apoptosis by XS-5 and XS-6 involves a caspase-dependent pathway. Expression of members of the Bcl-2 family was determined to identify the apoptotic signaling involved in HCC cells treated with XS-5 and XS-6. Mcl-1 is essential for maintaining mitochondrial membrane integrity and is involved in the release of cytochrome *c* following DNA damage [20]. XIAP, another antiapoptotic protein, has also been implicated in mitochondrial dysfunction and apoptosis [21]. Our study showed that XS-5 and XS-6 effectively diminished the elevated expression of Mcl-1 and XIAP. Moreover, JC-1 staining revealed that XS-5 and XS-6 induced marked changes in mitochondrial membrane potential and increased cytochrome *c* release from mitochondria. These results are consistent with previous reports that the aerial parts of XS induced apoptosis in HCC cells [22]. Our findings suggest that the induction of apoptosis by XS-5 and XS-6 could be associated with the caspase-dependent cascade, which involves the activation of the mitochondrial pathway initiated by the inhibition of Mcl-1 and XIAP. These events were supported by *ex vivo* results, showing that XS-5 and XS-6 increased the expression of cleaved caspase-3 and DNA fragmentation by TUNEL and led to apoptosis in tumor spheroids obtained from xenograft tumor tissues. In addition to the apoptosis effect, XS-5 and XS-6 also inhibited the migration and invasion of HCC cells. These results reveal that apoptosis, as well as the inhibition of cell growth and migration/invasion by XS-5 and XS-6, might contribute to the suppression of tumor growth.

Given that XS-5 and XS-6 induced apoptosis in HCC, we tried to find the main anticancer components of the underlying mechanisms. Unfortunately, the main components of XS-5 and XS-6 did not have better inhibitory effects on cell growth nor did they augment apoptosis in HCC cells (data not shown). Our results suggest that the various components of XS-5 and XS-6 could synergistically induce anticancer effects in HCC, which is characteristic of natural products.

The PI3K/AKT pathway is aberrantly activated in 30%–50% of HCC cases, which contributes to aggressive phenotypes and resistance to chemotherapy [23, 24]. Additionally, clinical studies have reported correlations between activation of the PI3K/AKT pathway, tumor progression, and reduced survival [25, 26]. Inhibition of PI3K/AKT signaling should, therefore, have strong anticancer effects against HCC. Although some types of XS have shown anticancer effects, studies on the anticancer mechanisms of XS are lacking. Accordingly, we attempted to investigate the effects of XS-5 and XS-6 on the PI3K/AKT pathway in HCC cells. As expected, XS-5 and XS-6 inhibited the phosphorylation of AKT and mTOR, downstreams of PI3K. Overall, these results indicate that XS-5 and XS-6 can both inhibit cell growth and induce apoptosis via regulation of the PI3K/AKT pathway.

## 5. Conclusion

Ethanol extracts of *Xanthium strumarium* (XS-5 and XS-6) exhibited potent anti-HCC activity via inhibition of the PI3K/AKT pathway, inhibiting cell proliferation and inducing apoptosis. Thereby, XS-5 and XS-6 could be used as a potential therapeutic agent for the treatment of HCC.

## Data Availability

The data used to support the findings of this study are available from the corresponding author upon request.

## Abbreviations

XS: *Xanthium strumarium*
HCC: Hepatocellular carcinoma
XIAP: X-linked inhibitor of apoptosis protein
PI3K: Phosphoinositide 3-kinase
PCNA: Proliferating cell nuclear antigen
mTOR: Mechanistic target of rapamycin.
